# Potential anti-hepatocellular carcinoma properties and mechanisms of action of clerodane diterpenes isolated from *Polyalthia longifolia* seeds

**DOI:** 10.1038/s41598-022-13383-y

**Published:** 2022-06-03

**Authors:** Vinay Bharadwaj Tatipamula, Chandi Vishala Thonangi, Tikam Chand Dakal, Girija Sastry Vedula, Bhanupriya Dhabhai, Haritha Polimati, Annapurna Akula, Ha Thi Nguyen

**Affiliations:** 1 Center for Molecular Biology, College of Medicine and Pharmacy, Duy Tan University, Danang, 550000 Vietnam; 2 Pharmacology Department, AU College of Pharmaceutical Sciences, Andhra University, Visakhapatnam, 530003 India; 3 Genome & Computational Biology Lab, Department of Biotechnology, Mohanlal Sukhadia University, Udaipur, Rajasthan 313001 India

**Keywords:** Biochemistry, Cancer, Chemical biology

## Abstract

Diterpenes are secondary metabolites that have attracted much attention due to their potential biological activities including anti-cancer potential. The aim of the current study is to assess the anticancer potential of the six known clerodane diterpenes (**1**–**6**) isolated from *Polyalthia longifolia* seeds and their underlying molecular mechanisms. These compounds were evaluated for their cytotoxicity in vitro by using MTT assays. The “two-phase model” with NDEA and PB ad libitum was used for induction of HCC and sorafenib was used as the standard drug. Prophylactic studies were carried out for compounds **4/6** at both low (5 mg/kg b.w) and high (10 mg/kg b.w) doses. Based on the MTT assay results, the two best compounds, **4** and **6**, were selected for in vivo studies. The results showed that treatment with compound **4/6** significantly restored the changes in biochemical parameters and liver morphology observed in (NDEA + PB)-induced HCC rats. Additionally, the docking studies showed that compound **4/6** interacted with several key proteins such as MDM2, TNF-α, FAK, thereby inhibiting these proteins and reversing the negative impacts of NDEA. In conclusion, our results suggested that compounds **4** and **6** are potential therapeutic agents for HCC, mostly due to their ability to control typical cancer pathways.

## Introduction

Hepatocellular carcinoma (HCC) is the most common form of primary liver cancer, accounting for 85% of all primary liver cancer cases^1^. HCC is characterized by a high rate of proliferation, metastasis, and invasiveness that are the key indicators for poor prognosis. Despite recent advances in diagnosis, a large proportion of liver cancer patients are diagnosed with advanced-stage tumors with no or very limited treatment options^2^. As a result, the survival rate of HCC patients is relatively low^3^, with the general 5-year survival rate ranging from ~ 8 to 18%^4–6^. Over the past three decades, the incidence of HCC has doubled and is predicted to increase until 2030^7^.

The liver plays a principal role in host defense and tumor transformation. Recent evidence suggests that inherent immunity is also important in the pathogenesis of liver fibrosis, providing new therapeutic targets for the treatment of different liver disorders^8^. However, inherent immunity can also precisely identify infection through pattern-recognition receptors (PRRs) that distinguish specific structures called pathogen-associated molecular patterns (PAMPs) conveyed by the invading pathogens^9^. Toll-like receptors (TLRs), a subgroup of PRRs, have been reported to be intricate in the activation of the transcription factor nuclear factor-kappa B (NF-kB) in hepatocytes^10^. NF-kB activation drives the production of pro-inflammatory cytokines, including IL-6, that in turn induce the stimulation of signal transducer and activator of transcription-3, thereby promoting hepato-carcinogenesis^11^.

Hepatocellular carcinogenesis is strongly associated with the induction of inflammation, mostly involves the release of IL-1α, which in turn promotes the expression of IL-6 and TNF^12^. Lower production of IL-6 in female due to the presence of estrogen, explained the male gender bias to HCC development^13^. To date, sorafenib, a multiple kinase inhibitor, is the only drug that has been approved by the US Food and Drug Administration for the treatment of HCC, suggesting an urgent need for new anti-HCC drugs, especially those with natural origin. Terpenoids are secondary metabolites, containing basic isoprene moiety with profound pharmacological activities. Previous studies have reported the use of terpenoids in the treatment of various cancers^14–16^.

The *Polyalthia longifolia* (Sonn.) Thwaites genus belongs to Annonaceae family. In Ayurveda, herbal preparations of *P. longifolia* have been mainly used to treat duodenal ulcers, cancer, fever, diabetes and skin diseases in various traditional medicine systems^14–18^. Furthermore, the bark and leaves of *P. longifolia* have been used to treat microbial infection, cancer, inflammation, diabetes and multiple diseases of the digestive system^17,18^. In the present study, we aimed to isolate the clerodane diterpenoids from seeds of *P. longifolia* plants and evaluate for their anticancer activity against *N*-nitrosodiethylamine (NDEA)-induced HCC.

## Results

### Isolation and identification of clerodane diterpenes

**Figure 1:**
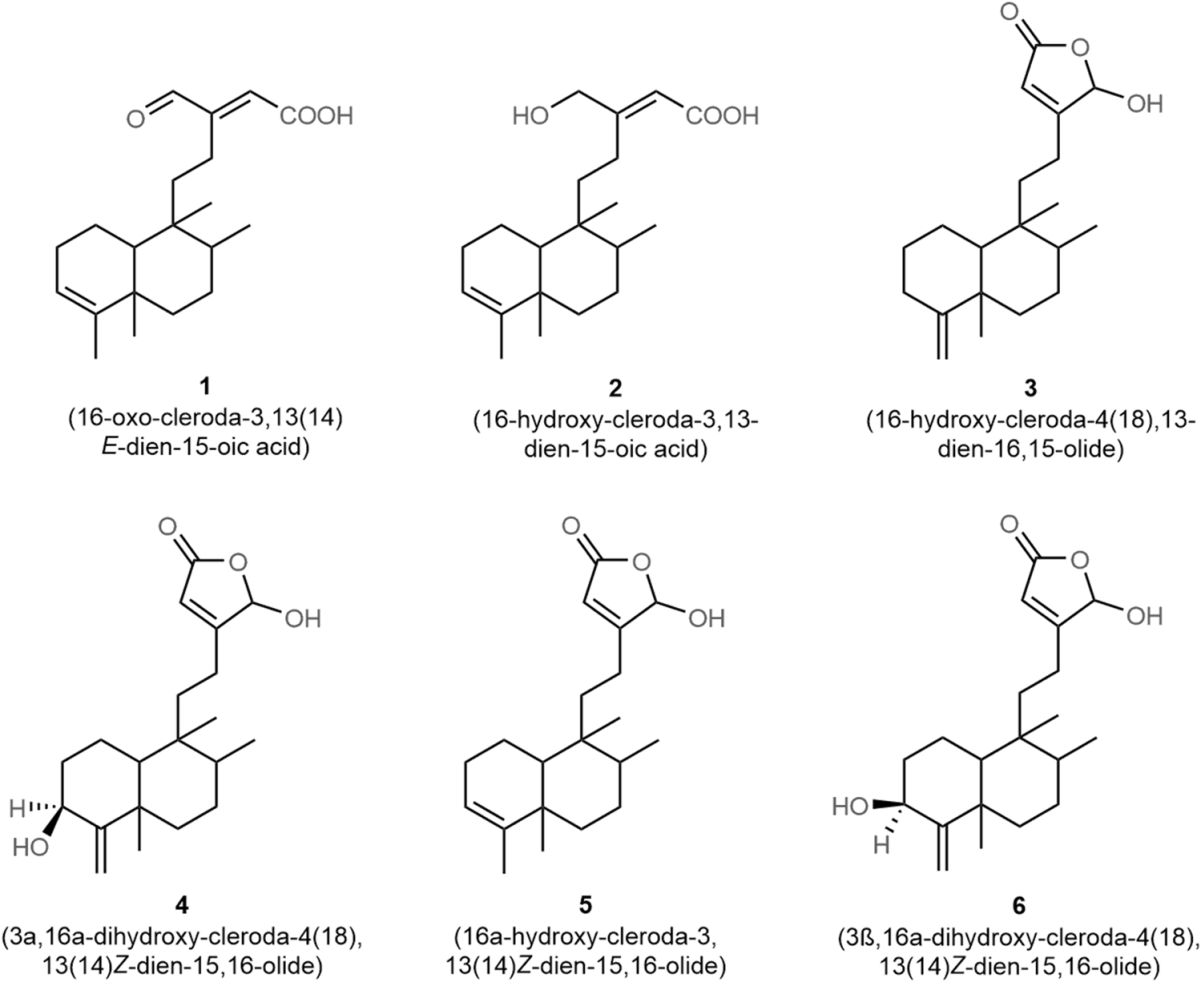
Chemical representation of clerodane diterpenes (**1**–**6**) from the *Polyalthia longifolia* (Sonn.) Thwaites seeds.

By employing chromatographic and spectral analytical methods, six known clerodane diterpenes (**1**–**6**) were identified from the methanolic extract of seeds of *P. longifolia*. The obtained spectral data were correlated with previous publications and the compounds were identified to be 16-oxo-cleroda-3,13(14)*E*-dien-15-oic acid (**1**)^17^, 16-hydroxy-cleroda-3,13-dien-15-oic acid (**2**)^17^, 16-hydroxy-cleroda-4(18),13-dien-16,15-olide (**3**)^17^, 3α,16α-dihydroxy-cleroda-4(18),13(14)*Z*-dien-15,16-olide (**4**)^17^, 16α-hydroxy-cleroda-3,13(14)*Z*-dien-15,16-olide (**5**)^17^, and 3β,16α-dihydroxy-cleroda-4(18),13(14)*Z*-dien-15,16-olide (**6**)^18^ (Figs. 1, S1).

### In vitro cytotoxicity of clerodane diterpenes

**Figure 2:**
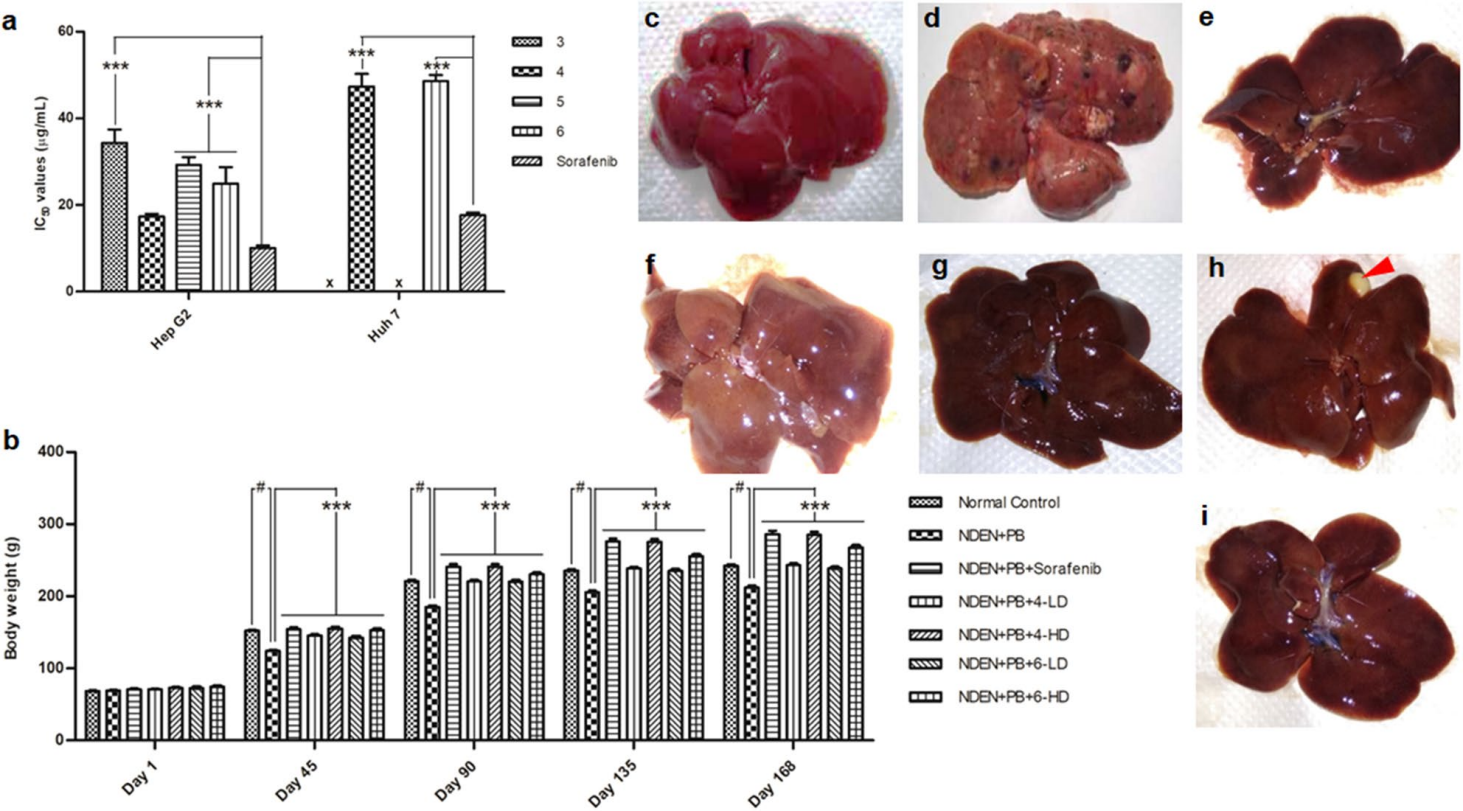
(**a**) Half-maximal inhibitory concentration (IC_50_) values of clerodane diterpenes against HepG2 and Huh 7 cancer cell lines. Those with IC_50_ values above 50 µg/mLwere considered as inactive and indicated with “x”. (**b**) The counteractive effect of **4** and **6** on body weight of (NDEA + PB)-induced hepatocellular carcinoma in Sprague–Dawley rats. Macroscopic features of livers from (**c**): Normal control; (**d**) Disease control (NDEA + PB); (**e**) Standard control (NDEA + PB + Sorafenib); and four tested groups: (**f**) NDEA + PB + 4-LD; (**g**) NDEA + PB + 4-HD; (**h**) NDEA + PB + 6-LD with a single nodule indicated by a red triangle; (**i**) NDEA + PB + 6-HD. NDEA: *N*-nitrosodiethylamine (200 mg/kg); PB: phenobarbital sodium (500 parts per million). Sorafenib: 30 mg/kg; **4**-/**6**-LD: 5 mg/kg; **4**-/**6**-HD: 10 mg/kg.

All the isolated clerodane diterpenes (**1**–**6**) were evaluated for their cytotoxicity against human hepatoblastoma (Hep G2) and human hepatoma-derived 7 (Huh 7) cancer cell lines. Among those, compounds **4** and **6** presented as the most potent cytotoxic agents against both Hep G2 cell line (IC_50_ values of 14.34 ± 0.65 and 24.91 ± 3.79 µg/mL, respectively) and Huh 7 cell line (IC_50_ values of 47.32 ± 2.94 and 48.57 ± 1.42 µg/mL, respectively) (Fig. 2a, Table S2). Compounds **3** and **5**, on the other hand, showed moderate cytotoxic activity against Hep G2 cell line only with IC_50_ values of 34.33 ± 3.08 and 29.21 ± 1.83 µg/mL, respectively (Table S2). However, all these IC_50_ values were found to be significantly higher than that of the standard drug, sorafenib, on both cell lines (Hep G2: 10.06 ± 0.61 µg/mL and Huh 7: 17.61 ± 0.62 µg/mL).

### Changes in body weight and liver morphometric parameters

All animals in the study showed a gradual weight gain throughout the course of the study. However, the disease control animals gain significantly less compared to the normal control groups (*P* < 0.001). The standard control and all four tested groups showed significant differences in the weight gain compared to the disease control group (*P* < 0.001) (Fig. 2b).

At the end of the study, all the rats were sacrificed and their livers were collected to weigh and do a morphology check. The disease control ((NDEA + PS)-treated) group appeared to have multiple tumors and a significant increase in liver weight compared to the normal control group (Fig. 2c,d), which was significantly recovered in the standard control (Fig. 2e) and all the **4**-/**6**-treated (Fig. 2f–i) groups. Particularly, in the treated groups, only a single nodule was observed in the **6**-LD-treated group (Fig. 2h), whereas in the remaining groups, formation of nodules was not observed.

### Compounds **4** and **6** reversed the negative impacts on biochemical parameters of the HCC rats

**Figure 3:**
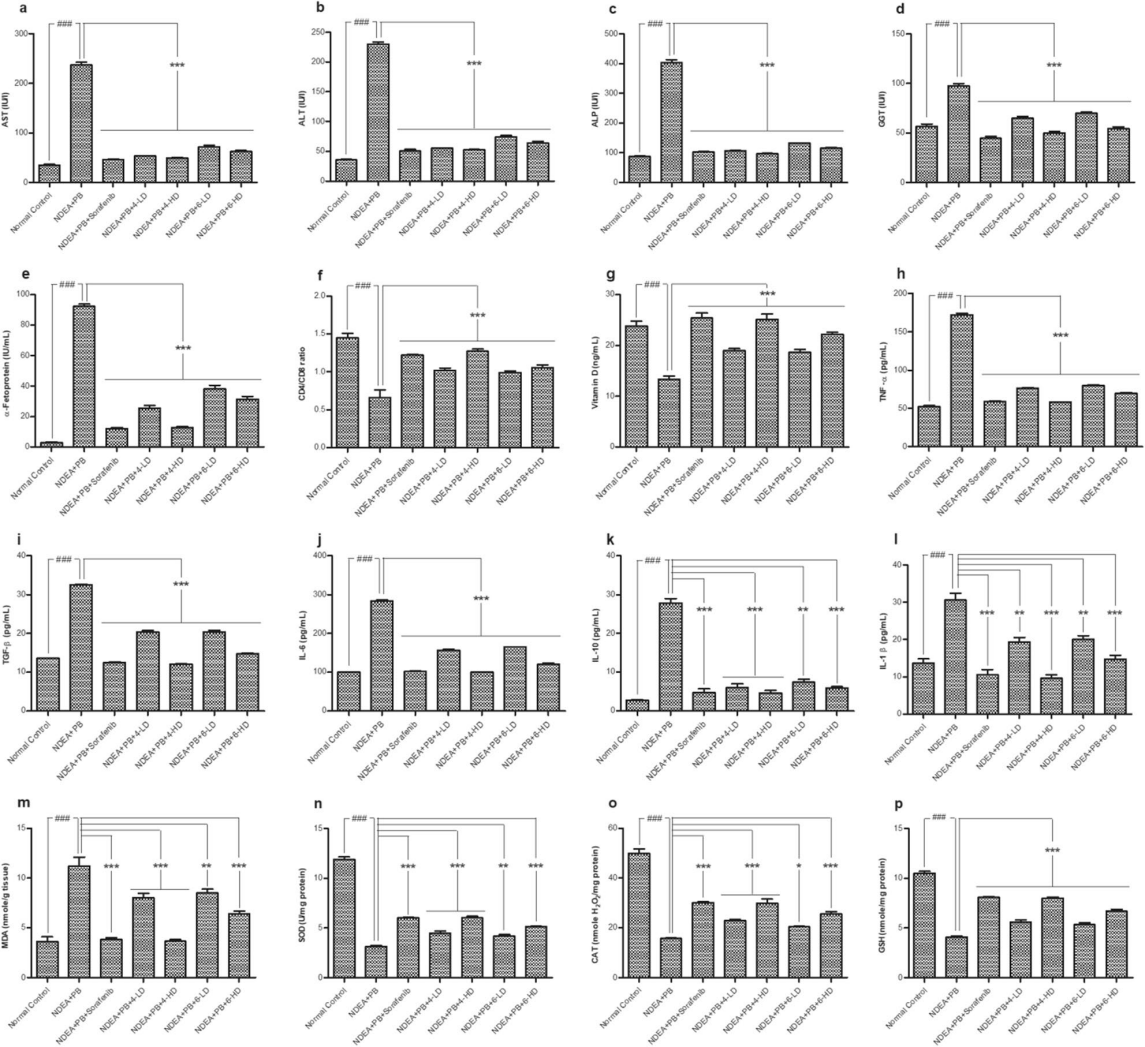
Effect of **4** and **6** on biochemical parameters of (NDEA + PB)-induced hepatocellular carcinoma in Sprague–Dawley rats. (**a**) Aspartate aminotransferase (AST) levels; (**b**) Alanine aminotransferase (ALT) levels; (**c**) Alkaline phosphatase (ALP) levels; (**d**) Gamma glutamyl transpeptidase (GGT) levels; (**e**) α-Fetoprotein; (**f**) CD4/CD8 ratio; (**g**) Vitamin D; (**h**) Tumor necrosis factor-α (TNF-α); (**i**) Tissue growth factor-β (TGF-β); (**j**) Interleukin-6 (IL-6); (**k**) Interleukin-10 (IL-10); (**l**) Interleukin-1 β (IL-1β); (**m**) Malondialdehyde (MDA); (**n**) Superoxide dismutase (SOD); (**o**) Catalase (CAT); (**p**) Glutathion reductase (GSH). All the values were expressed as mean ± standard error of the mean where *n* = 10 animals. The data were analyzed by one-way analysis of variances, followed by Tukey’s test, where *P < 0.05, **P < 0.01, and ***P < 0.001 statistically significant when compared with the disease control, and ^###^*P* < 0.001 statistically significant when compared with normal control. The values in the parentheses indicate the percentage protection. NDEA: *N*-nitrosodiethylamine (200 mg/kg); PB: phenobarbital sodium (500 parts per million). Sorafenib: 30 mg/kg; **4-/6-**LD: 5 mg/kg; **4-/6-**HD: 10 mg/kg.

The disease control rats had a significant increase in liver function parameters like aspartate aminotransferase (AST), alanine aminotransferase (ALT), alkaline phosphatase (ALP) and gamma-glutamyl transpeptidase (GGT) as compared to the normal control (*P* < 0.001). The aberration of liver function parameters in the (NDEA + PB)-treated rats was significantly reversed upon administration with compound **4** or **6** in a time- and dose-dependent manner. Among four tested groups, rats administered with **4-**HD presented as the most protected ones, with liver function parameters similar to that of the standard group (Fig. 3a–d).

Additionally, (NDEA + PB) intoxication was significantly (*P* < 0.001) elevated in α-fetoprotein (AFP) levels (92.30 ± 1.41 IU/mL) in comparison to the normal control (2.76 ± 0.41 IU/mL). However, this effect was significantly (*P* < 0.001) reversed in rats treated with **4** or **6** in a dose-dependent manner (Fig. 3e). Of these, **4-**HD appeared to give the best protective effect, with AFP levels (12.80 ± 0.67 IU/mL) found to be similar to that of the standard (12.19 ± 0.62 IU/mL) and the normal control (12.76 ± 0.41 IU/mL) (Fig. 3e). The CD4/CD8 ratio and the levels of vitamin D, however, were significantly decreased in the disease control group (*P* < 0.001); these were significantly attenuated by **4** or **6** in a dose-dependent manner (*P* < 0.001) (Fig. 3f). Specifically, **4-**HD was able to recover the CD4/CD8 ratio (1.27 ± 0.03) and vitamin D levels (25.14 ± 1.01 ng/mL) in the disease control to a level similar to that of the standard (CD4/CD8: 1.22 ± 0.01, vitamin D: 25.39 ± 0.98 ng/mL) and the normal control (CD4/CD8: 1.45 ± 0.06, vitamin D: 23.78 ± 1.04 ng/mL) groups (Fig. 3f,g).

### Compounds **4** and **6** restored the levels of pro-inflammatory markers and oxidative stress markers in the HCC rats

The (NDEA + PB) intoxication significantly (*P* < 0.001) raised all five tested pro-inflammatory proteins in the disease control as compared to the normal control group, with fold changes for the levels of TNF-α, TGF-β, IL-6, IL-10, and IL-1 noted to be approximately 3.3, 2.4, 2.9, 9.9, and 2.3, respectively (Fig. 3h–l). However, these changes were also significantly reversed by administration of compound **4** or **6**. Among all the tested conditions, **4-**HD also showed to be the most potent in reversing the negative effects caused by (NDEA + PB) and brought the levels of all five pro-inflammatory proteins to a level similar to that of the standard and normal control groups (Fig. 3h–l).

Lipid peroxidation (malondialdehyde, MDA) and antioxidant levels were significantly (*P* < 0.001) altered in the disease control as compared to the normal control group (Fig. 3m). Specifically, MDA was increased > threefold while the antioxidants catalase (CAT), superoxide dismutase (SOD), and reduced glutathione (GSH) were decreased ~ 3.8-, 3.2-, and 2.6-fold in the disease control versus normal control rats (Fig. 3m–p). Interestingly, these aberrations were abolished partly or completely by administration of **4** or **6**. Again, **4-**HD presented as the best condition to reverse the changes observed in the disease control group to the values close to that of the standard control and normal control for MDA, and a bit lower than that of the normal control for antioxidant parameters (Fig. 3m–p).

### Compounds **4** and **6** restored the liver histopathological properties in the HCC rats

**Figure 4:**
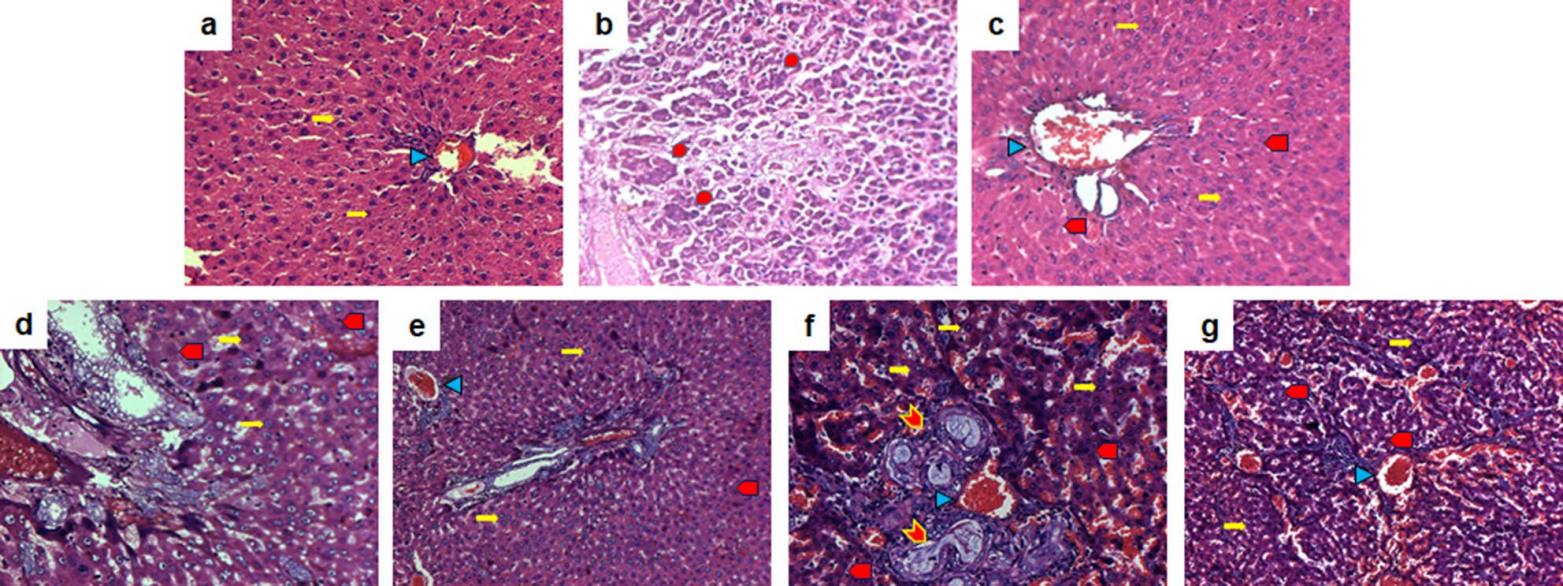
Effect of compounds **4** and **6** on histopathological properties of (NDEA + PB)-induced hepatocellular carcinoma in Sprague–Dawley rats at 100x. (**a**): Normal control; (**b**) Disease control (NDEA + PB); (**c**) Standard control (NDEA + PB + Sorafenib); and four tested groups: (**d**) NDEA + PB + **4**-LD; (**e**) NDEA + PB + **4**-HD; (**f**) NDEA + PB + **6**-LD; (**g**) NDEA + PB + **6**-HD. NDEA: *N*-nitrosodiethylamine (200 mg/kg); PB: phenobarbital sodium (500 parts per million). Sorafenib: 30 mg/kg; **4-/6-**LD: 5 mg/kg; **4-/6-**HD: 10 mg/kg. Yellow right arrow: Healthy hepatocytes; Blue isosceles triangle: central vein; Red teardrop: fibrosis and cytoplasmic fat infiltration; Red pentagon: regeneration of necrotic hepatocytes; Red chevron: tumor nodules.

The liver tissues of the control rats showed normal morphology of the central vein, normal sinusoids, healthy hepatocytes with round nuclei and granulated cytoplasm, and no infiltration of inflammatory cells (Fig. 4a). Liver tissues from the NDEA-induced HCC rats, on the other hand, showed loss of typical liver morphology with fibrosis and cytoplasmic fat infiltration; changes in nucleus shape and size of hepatic cells, for example irregular nuclear membrane pattern, coarsely granular chromatin, and abnormal mitotic figure; severe hepatic dysplasia with intrahepatic vein; and numerous large tumor cells (Fig. 4b). These effects, however, were significantly recovered in the standard control (Fig. 4c) and all the treated groups (Fig. 4d–g). The **4-**LD**, 6-**LD, and **6-**HD-treated groups showed a regain of typical liver architecture at a certain level, with less hepatic dysplasia and intrahepatic vein (Fig. 4d,f,g). However, there were still tumor nodules in the **6**-LD (Fig. 4f) and sinusoids with a focal area of mild inflammatory cell aggregates with normal hepatocyte features in the **6**-HD-treated groups (Fig. 4g). HCC rats administered with **4-**HD (Fig. 4e) and standard sorafenib (Fig. 4c), on the other hand, recovered normal liver cell and tissue architecture to a level similar to that of the normal control.

### Molecular docking analysis

Molecular docking of compounds **4** and **6**, the cancer-inducing agent NDEA, and the standard sorafenib was performed on the targeted proteins, namely MDM2, TNF-α, TGF-β, FAK, and IL-6. The binding affinities (kcal/mol) were obtained for all the ligands (Table 1).

**Table 1:** Hydrogen bonds and binding affinities of the protein–ligand docked complexes.

Protein	Hydrogen bonds and binding affinity (kcal/mol)							
	NDEA		Sorafenib		**4**		**6**	
MDM2	Cys49	− 3.6	Gln44	− 7.5	Leu54	− 6.5	Gly58	− 6.7
TNF-α	Glu87, Asp113	− 3.6	Lys49, Leu112	− 7.5	Gln61	− 6.2	Leu120	− 6.1
TGF-β	Tyr173, Ala175, Ala193	− 4.7	Arg129, Gly169, Asp180	− 7.5	–	–	Glu284	− 6.1
FAK	Leu146	− 3.3	Arg159	− 7.2	Lys454	− 7.1	Lys454	− 7.2
IL-6	–	–	Leu84	− 7.6	Ser156, Gln158	− 5.8	–	–

**Figure 5:**
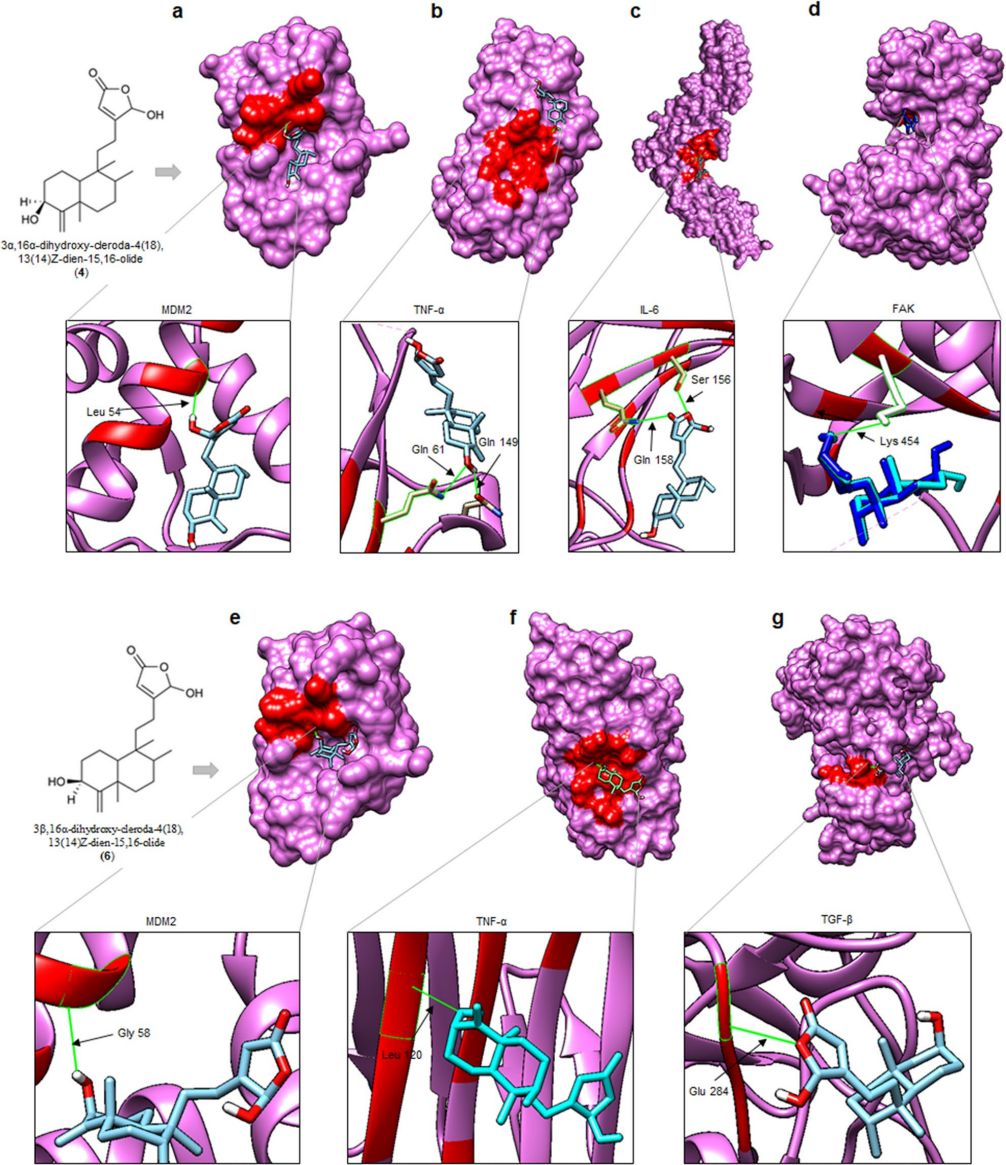
Interaction between compounds **4** and **6** and the target proteins. The 3D interaction and hydrogen bonds between (**a**) **4**-MDM2, (**b**) **4**-TNF-α, (**c**) **4**-IL-6, and (**d**) **4/6**-FAK, (**e**) **6**-MDM2, (**f**) **6**-TNF-α, (**g**) **6**-TGF-β. Hydrogen bond interaction between proteins and ligand shown in green color, and receptor active sites shown in red color.

The docking results showed that compound **4** docked into the active sites of MDM2, TNF-α, FAK, and IL-6; while compound **6** docked into the active sites of MDM2, TNF-α, TGF-β, and FAK. Specifically, compound **4** formed hydrogen bonds with Leu54 of MDM2; Gln61, Gln149 of TNF-α; Lys454 of FAK; and Ser156, Gln158 of IL-6 with a binding affinity of − 6.5, − 6.2, − 7.1, and − 5.8 kcal/mol, respectively (Table 1, Fig. 5). On the other hand, compound **6** interacted and formed a hydrogen bond with Gly58 of MDM2; Leu120 of TNF-α; Lys454 of FAK; and Glu284 of TGF-β, with a binding affinity of − 6.7, − 6.1, − 7.2, and − 6.1 kcal/mol, respectively (Table 1, Fig. 5).

Both NDEA and sorafenib interacted with the studied proteins via hydrogen bonds and hydrophobic interactions (π-π stacking). Particularly, the NDEA molecule formed hydrogen bonds with Cys49 of MDM2; Glu87, Asp113 of TNF-α; Tyr173, Ala175, Ala193 of TGF-β; and Leu146 of FAK (Table 1, Fig. S2). Sorafenib, on the other hand, formed hydrogen bonds with Gln44 of MDM2; Lys49, Leu112 of TNF-α; Arg129, Gly169, Asp180 of TGF-β; Arg159 of FAK; and Leu84 of IL-6 and hydrophobic interactions with His67 of MDM2; and Tyr111, Phe114 of TNF-α (Table 1, Fig. S3).

## Discussion

**Figure 6:**
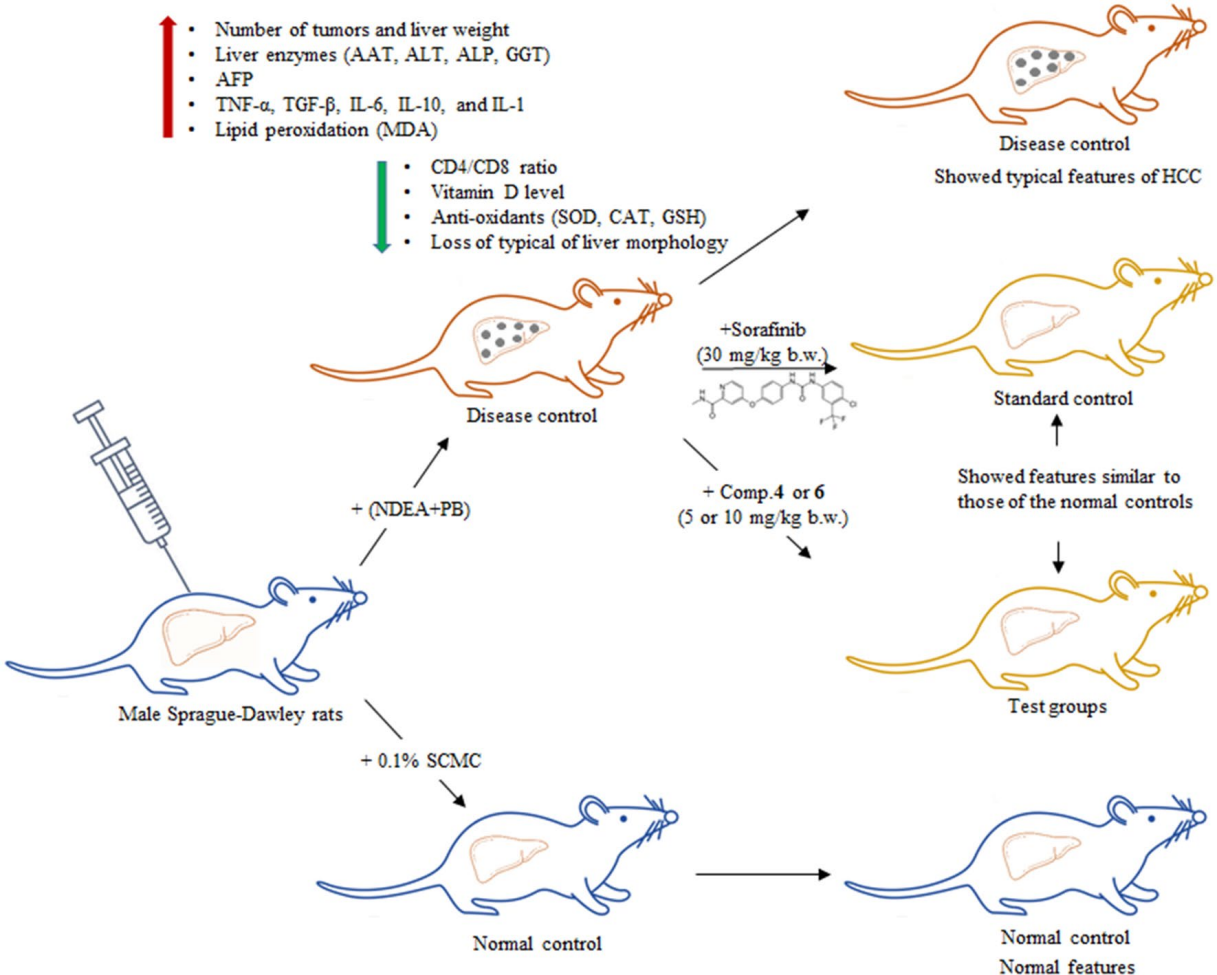
General experimental procedure and the key protective effects of compounds **4** and **6** against (NDEA + PB)-induced HCC in rats. HCC: hepatocellular carcinoma; SCMC: sodium carboxymethyl cellulose; NDEA: *N*-nitrosodiethylamine; PB: phenobarbital sodium; Comp.: compound; b.w: body weight.

The liver carcinogenicity of NDEA has been widely reported and the two-stage model NDEA + PB is commonly used to generate HCC models^19,20^. NDEA mainly undergoes metabolic biotransformation by cytochrome p450 enzyme and produces toxic active metabolites involved in the formation of DNA adducts. This in turn results in the generation of reactive oxygen species (ROS), causing oxidative stress and DNA damage, and eventually initiating the development of preneoplastic and dysplastic nodules^21^. In the present study, all the toxicant-induced animals developed liver tumors followed by an increased liver weight, indicating the success of the induction model. The administration of the disease control rats with compound **4** or **6** significantly reduced the tumor burden and liver weight in a dose-dependent manner. These findings are in accordance with previous studies that reported the cytotoxic and genoprotective properties of terpenoids of *P. longifolia*^22,23^. Conversely, the body weights of the disease control rats were significantly reduced as compared to the normal control rats. This observation can be explained by the fact that the liver is the major site for metabolism; thus, damage in liver tissues due to the hepatocarcinogenic property of the NDEA may cause loss of appetite and a decrease in adipogenesis and food ingestion, and eventually low body mass^24^. The study design and the key findings of the present study was presented in Fig. 6.

The assessment of the liver parameters showed a significant increase in the levels of liver enzymes ALT, AST, ALP, and GGT in the disease control as compared to both the normal control and **4**-/**6**-treated groups. This might be due to an effect of NDEA + PB that causes loss of the membrane integrity, leading to a spill of liver enzymes and raising their serum levels^25^. Of these enzymes, ALT, AST, and ALP are known as sensitive indicators of hepatic damages^26^ while GGT, a membrane-bound enzyme, is an indicator for changes in physiological conditions, including hepatocarcinoma^27^. The **4-/6**-treated groups, however, had significantly reversed levels of these liver enzymes, to a level similar to that of the normal control group (Fig. 3a–d). This finding strongly indicated the protective effects of these clerodane diterpenes against (DEN + PB)-induced liver tumor formation and progression in rats, possibly via reduction of ROS generation and restoring DNA repair mechanisms^28^.

Additionally, biochemical parameters such as the levels of AFP and vitamin D and CD4/CD8 ratio were accessed. In the present study, AFP enzyme presented as highly induced in the disease control as compared to the normal control rats, which was then significantly reversed in the **4**-/**6**-treated rats in a dose-dependent manner (Fig. 3e,f), suggesting potential antitumor properties of these compounds^29,30^. AFP is a 70 kDa glycoprotein that is synthesized by embryonic cells of the yolk sac in the fetal liver and degenerated in the adult liver^31^. AFP plays a major role in the development of HCC and is largely considered as an immunotherapy target for HCC^32^ on one hand, AFP promotes tumor proliferation via induction of mitosis, invasion, and angiogenesis of tumor cells^33^; on the other hand, it suppresses apoptosis through activation of the PI3K/Akt/mTOR signaling pathway^34^.

In contrast, the CD4/CD8 ratio and the level of vitamin D were found to be significantly decreased in disease control rats, which was reversed in the **4**-/**6**-treated groups. Vitamin D is a fat-soluble vitamin, mainly involved in maintenance of calcium homeostasis, proliferation, differentiation, inflammation, and immune modulation^35^. The increased levels of vitamin D in the **4**-/**6**-treated groups indicated that the clerodane diterpenes might have upregulated the vitamin D receptors in the membrane of liver cells, thereby increasing the vitamin D uptake and its serum levels^36^. Chronic hepatic disorders might cause abnormal expression of vitamin D receptors resulting in a decrease in serum levels of vitamin D, which has been linked to poor survival rates^37^ and an increased risk of hepatic disorders^38^. Vitamin D supplements, on the other hand, inhibit COX expression and reduce pro-inflammatory cytokines such as IL-6, IL-10, and TNF-α, while expressing antioxidant properties by increasing endogenous antioxidants and inhibiting oxidative stress-induced DNA damage. Therefore, vitamin D supplements may be considered as a potential approach in HCC control.

Cytokines are the major components of the immune system involved in tumor initiation and progression. In response to inflammation, pro-inflammatory cytokines such as TNF-α, TGF-β, IL-10, IL-6, IL-8, and IL-1β are synthesized^39,40^, and their increased levels have been identified as an indicator of the pathogenesis of HCC^41^ and other solid tumors^42^. In our study, all these pro-inflammatory cytokines were significantly increased in the disease control group, and their levels were substantially reversed to a level similar to that of the normal control after administrating with **4** or **6**, suggesting the potential anti-cancer properties of these two compounds. These results were in concordance with previous studies of the anti-inflammatory properties of clerodane diterpenes isolated from another plant, *P. longifolia*^17,43^. TGF-β was known to induce the differentiation of CD4+ T cells into regulatory T cells that, in turn, play an essential role in the progression of HCC^44^. Thus, inhibition of the TGF-β signaling pathway may also be a potential approach for anti-HCC therapy^44^.

Increased levels of free radicals due to inflammation initiate the oxidation of polyunsaturated fatty acids, leading to lipid peroxidation and the production of MDA^45^. Increased levels of MDA in turn lead to oxidative stress, which is known as a causative factor of carcinogenesis^46^. The excessive generation of ROS leads to an imbalance between reactive species and antioxidants, resulting in a decrease of antioxidant enzymes like SOD, CAT, and GSH, and eventually in tumor growth^47^. In the present study, the administration of (NDEA + PB) significantly increased MDA and decreased antioxidant activity, whereas the **4**-/**6**-treatment groups significantly attenuated the effects. These activities may be attributed to the antioxidant and anti-inflammatory properties of *P. longifolia*^48^.

The molecular docking results were analyzed on the basis of binding affinity. In Autodock Vina, the docking score indicates the binding affinity of protein–ligand interactions in kcal/mol. A lower docking score shows a higher binding affinity^49^. Accordingly, the standard sorafenib has the highest binding affinity, followed by compounds **4** and **6**, and then NDEA with the lowest binding affinity to all five tested proteins, MDM2, TNF-α, TGF-β, FAK, and IL-6.

NDEA is a carcinogenic agent that is commonly used in experimental research to induce HCC^50^. The continuous generation of ROS in the liver induced by NDEA in vivo causes oxidative stress and eventually damages biological systems, which are involved in all steps of carcinogenesis^51^. During tumorigenesis, different pro-inflammatory cytokines such as IL-6, IL-10, TNF-α, and TGF-β are upregulated, thereby promoting inflammation and carcinogenesis. In the presence of compounds **4, 6,** or sorafenib, these ligands bind to the active site of these proteins, causing protein inactivation, thereby reducing or reversing the negative impacts of NDEA. MDM2, on the other hand, promotes tumor formation by targeting tumor suppressor protein P53 for proteasomal degradation^52^.

Additionally, the docking results showed a good binding affinity of these compounds to the MDM2 protein, suggesting that anti-cancer properties of these compounds may be achieved via MDM2/P53 axis. Inhibiting the P53-MDM2 interaction has been recognized as an important target for cancer therapy^53^. FAK was identified as overexpressed and was associated with aggressiveness in adult HCC patients^54^. Inhibition of FAK decreases HCC invasiveness by down-regulating EZH2, an epigenetic controller that represses transcription of a large number of genes via H3K27me3^55^. The docking results of the present study showed a strong affinity of sorafenib, **4** and **6** to FAK. Possibly, the binding of these compounds to the active site of FAK makes this protein unable to bind the targets, thus reversing its impact on HCC progression.

Histopathological studies noted the gross morphological changes in liver tissue. Notably, the livers of the control (Fig. 4a), sorafenib-supplemented (Fig. 4c), and **4-**HD**-**supplemented (Fig. 4e) rats were found to have a normal architecture, whereas livers of the (NDEA + PB)-treated rats showed severe hepatic dysplasia with pyknotic nucleoli and cells exhibiting intra-nuclear, cytoplasmic vacuoles with cellular fat infiltration (Fig. 4b). The findings showed that the disease phenotypes were substantially suppressed in the **4-/6-**treated rats (Fig. 4d–g) with a regain of typical architecture and less hepatic dysplasia, which may be due to the anti-carcinogenic effect of clerodane diterpenes. However, among all the treated groups, only **4-**HD**-**supplemented rats presented to have better recovery in histological observations.

Together, our results indicated that compounds **4** and **6** may have strong anti-cancer properties that confer their effects either by directly interacting with cytokines (IL-6, IL-10), TNF-α, and TGF-β or via different axes such as MDM2/P53 and/or FAK/EZH2. The future in vitro studies on human hepatic cancer cell lines evaluating the mRNA and the protein expression levels of the identified targets followed the administration of the corresponding compounds (NDEA, Sorafinib, **4**, or **6**) are necessary to confirm the findings of the present study and further specify the underlying molecular pathways of these two clerodane diterpenes.

## Conclusions

In summary, the present study showed that treatment with compounds **4** or **6** significantly enhanced the biochemical parameters including liver enzymes, Vitamin-D level, CD4/CD8 ratio, and the level of antioxidant enzymes; while significantly decreasing the level of lipid peroxidation and pro-inflammatory cytokines in a dose-dependent manner in (NDEA + PB)-induced HCC rats. The docking studies indicated that these two compounds were able to bind and form hydrogen bonds with amino acid residue(s) within the active site of the carcinogenic proteins MDM2, TNF-α, FAK, IL-6, and TGF-β, causing protein inactivation and thereby inhibiting carcinogenesis induced by NDEA and PB. Taken together, the present study suggested that clerodane diterpenes **4** and **6** can be considered as candidates in the treatment of HCC. Further studies exploiting deeper mechanism of action and additional pre-clinical studies of these clerodane diterpenes would contribute to the fight against HCC.

## Methods

### Chemicals

NDEA, MTT (3-(4,5-dimethyl-thiazol-2-yl)-2,5-diphenyltetrazolium bromide), and Phenobarbital sodium (PB) were procured from Sigma Aldrich, St. Louis, USA. All other chemical agents used in this experiment are at analytical grade.

### Plant material

The seeds of *P. longifolia* were collected from Seshachalam Hills, Tirupati, India, in 2019. The sample has been authenticated and a voucher specimen (PS-2019-349) has been deposited at the Department of Botany, Sri Venkateswara University, India. The collection of plant material was complied with relevant institutional, national, and international guidelines and legislation.

### Extraction, isolation, and identification of compounds

About 1.0 kg of dried seed powder of *P. longifolia* was extracted with methanol using procedures as previously described^1^, and subjected to column chromatography. The chemical examination of obtained methanolic extract (40.0 g) yielded six known clerodane diterpenes (**1**–**6**) (Fig. 1)^17^.

### In vitro cytotoxic activity

The cytotoxic activities of all the isolated compounds (**1**–**6**) were identified via MTT assays on Hep G2 and Huh 7 cell lines (National Centre For Cell Science Pune, Mumbai, India) as previously described^56^.

### Preparation of test compounds, carcinogens, and induction of HCC

Two clerodane diterpenes (**4** and **6**) with the best in vitro cytotoxic activity were subjected to further in vivo study. Compounds **4** and **6** were made into a suspension by using 0.2% sodium carboxymethyl cellulose (SCMC) to obtain working solutions of 5 and 10 mg/mL.

The NDEA was diluted in 0.9% normal saline to obtain a working solution of 0.95 mg/mL and PB was diluted in purified water to obtain a concentration of 500 parts per million (ppm) and supplemented ad libitum*.*

The “two-phase model” employed a genotoxic compound, NDEA, at a single dose of 200 mg/kg body weight (b.w.) by intraperitoneal route in the initiation phase on day 10 of the treatment schedule. From the 2nd week onwards an anti-epileptic drug, PB 500 ppm, was administered ad libitum until the end of the study (24 weeks/168 days) to promote HCC^57^.

### Animals

Male Sprague–Dawley rats at 35 days of age, weighing from 25 to 35 g and Swiss albino mice weighing from 18 to 25 g were obtained from National Institute of Nutrition Laboratories, Hyderabad, Telangana, India. The animals were acclimatized and maintained in standard laboratory conditions (temperature: 25 ± 2 °C; relative humidity: 44–55%; 12-h light), standard diet (Krish Scientist Shoppe, India) and ad libitum water. The study protocols were in accordance with the guidelines of the Committee for the Purpose of Control and Supervision of Experiments on Animals and the study was approved by the Institutional Animal Ethical Committee of AU College of Pharmaceutical Sciences (Regd. No. 516/PO/c/01/IAEC/1).

### Acute toxicity studies

Toxicity studies were performed to determine the dose of the test compounds. The studies were carried out according to the OECD guidelines (Section 4 Test No. 423). Initially, the animals were fasted overnight and a single bolus dose of test substances at 5 mg/kg, 50 mg/kg, 300 mg/kg and 2000 mg/kg b.w. was administered to each group of 3 healthy mice by oral gavage. These mice were observed carefully for signs of toxicity, behavioral changes, and mortality in the first 2 h and continued up to 14 days^58^. Based on the results, the low (5 mg/kg b.w.) and high (10 mg/kg b.w.) doses were selected for further studies.

### Research design

Rats were equally and randomly divided into seven groups (*n* = 10) and different treatment procedures were applied. Of these, Group 1 served as normal control (0.1% SCMC); Group 2 served as disease control (NDEA + PB); Group 3 served as standard control (NDEA + PB + Sorafenib; and Group 4, 5, 6, and 7 served as test groups. Rats in the four test groups were pre-treated with NDEA and PB, followed by either **4** or **6** at low (5 mg/kg b.w.) or high (10 mg/kg b.w.) dose and were named as NDEA + PB + 4-LD, NDEA + PB + 4-HD, NDEA + PB + 6-LD, NDEA + PB + 6-HD, respectively.

Briefly, on day 10 of the treatment, all the groups except Group 1 were treated with DEN (200 mg/kg b.w.) through intraperitoneal route. Two weeks after DEN induction, PB (500 ppm) was administered with water ad libitum and continued until the end of the study. On day 40, treatment was given through oral gavage for Group 3 (Sorafenib, 30 mg/kg b.w.) and all the test groups (Groups 4–7), daily once in the evening (5.00 pm) until day 168.

### Evaluation of body weight and liver morphometric parameters

The body weight of animals in all groups was evaluated at a regular time interval of 15 days throughout the study period (24 weeks). At the end of the study, the animals were sacrificed and the livers were excised for evaluation of tumor incidence and tumor multiplicity.

### Evaluation of liver function and biochemical parameters

Multiple serum liver function parameters like alanine aminotransferase (ALT), aspartate aminotransferase (AST), alkaline phosphatase (ALP), and gamma-glutamyl transpeptidase (GGT) were evaluated by using respective diagnostic kits (Excel diagnostics Pvt. Ltd., Hyderabad, India) following the manufacturer’s instruction.

Serum biochemical parameters like α-fetoprotein (AFP) (Cat. No. ER0074; Biotrend CliniScineces Group) and vitamin D (Cat. No. ER0898; Universal Biologicals, Life Science Research Products) were evaluated by using Enzyme-linked Immunosorbent assay (ELISA) kits, while the ratio of CD4/CD8 T cells was assessed in flow cytometry applications using the BD Simultest™ CD4/CD8 (Cat. No. 340039, BD Biosciences).

### Evaluation of pro-inflammatory cytokines and oxidative stress parameters

At the end of the study, pro-inflammatory cytokines like TNF-α, TGF-β, IL-6, IL-10, and IL-1β (Assaypro, USA) were evaluated by following the manufacturer’s procedures.

Endogenous antioxidant parameters like catalase (CAT), superoxide dismutase (SOD), and reduced glutathione (GSH), and malondialdehyde (MDA) levels were measured as previously described^59^.

### Histopathology studies

Animals were sacrificed and the livers were harvested. The intact liver tissue was stored in formalin (10% v/v). The stored tissue samples of approximately 3 mm thickness were then paraffin-embedded. Tissue sections of 5–10 μm thickness were prepared using a microtome (Leica RM2125 RTS, Leica Biosystems, US) and stained with hematoxylin and eosin as previously described^60^. The stained slides were then examined for pathological changes by using a confocal laser scanning microscope (Zeiss LSM710, Germany) at a magnification of 100×.

### Statistical analysis

Data were presented as mean ± standard error of the means (SEM) where *n* = 10. Data were analyzed by using one-way analysis of variances (ANOVA) followed by Tukey’s test by using Graphpad Prism software (5.0 v) when compared to the disease control group. *P* values of less than 0.05 were considered statistically significant.

### Preparation of target proteins and ligands

The crystal structure of target proteins such as MDM2, TNF-α, TGF-β, FAK, AFP, and IL-6 was retrieved from the RCSB website (https://www.rcsb.org/). Protein data bank (PDB) structure of the AFP was not available online, which was therefore generated using I-TASSER tool (https://zhanggroup.org/I-TASSER/)^61^. This tool allows users to generate high-quality 3D structure models using state-of-the-art algorithms based on ab-initio modeling. Prior to docking, protein structures were prepared by removing water molecules using UCSF Chimera software^62^. Both ligand SMILES were converted into PDB files using the online SMILES Translator tool (cactus.nci.nih.gov/translate)^63^. The active site of the proteins was predicted from literature reviews as shown in Table S1.

### Protein–ligand molecular docking

Molecular docking was executed using AutoDock Vina, which is standalone software^64^. In the docking preprocessing step, the PDB format of protein and ligand molecules are converted into Autodock’s PDBQT format. From the AutoDock Vina, the protein molecule was modified with Gasteiger Partial charges and further hydrogen was also added. The protein–ligand docking was focused on the specified binding site. The grid box was defined within the binding sites of the protein structures with the configurations of x/y/z coordinates. The binding affinities between the receptors and the ligands were attained in terms of kcal/mol. The interaction of the compound with proteins and the formation of a hydrogen bond between the two molecules were visualized by UCSF Chimera.

### Statement

All methods were carried out and reported in accordance with ARRIVE guidelines (https://arriveguidelines.org) for the reporting of animal experiments and animal sacrificed during study.

## Supplementary Information

Supplementary Information.

## Data Availability

All data generated or analyzed during this study are included in this published article and its supplementary information files.
