# Supplementary material for: Potential anti-hepatocellular carcinoma properties and mechanisms of action of clerodane diterpenes isolated from Polyalthia longifolia seeds
Source: Sci Rep. 2022 Jun 3;12:9267. doi: 10.1038/s41598-022-13383-y (PMC9166726; doi:10.1038/s41598-022-13383-y)
Supplement: Supplementary file 1 — Supplementary Information. [file 41598_2022_13383_MOESM1_ESM.doc]

**Supporting information to**

**Potential anti- hepatocellular carcinoma properties and mechanisms of action of clerodane diterpenes isolated from *Polyalthia longifolia* seeds**

**Supplementary figures**

**
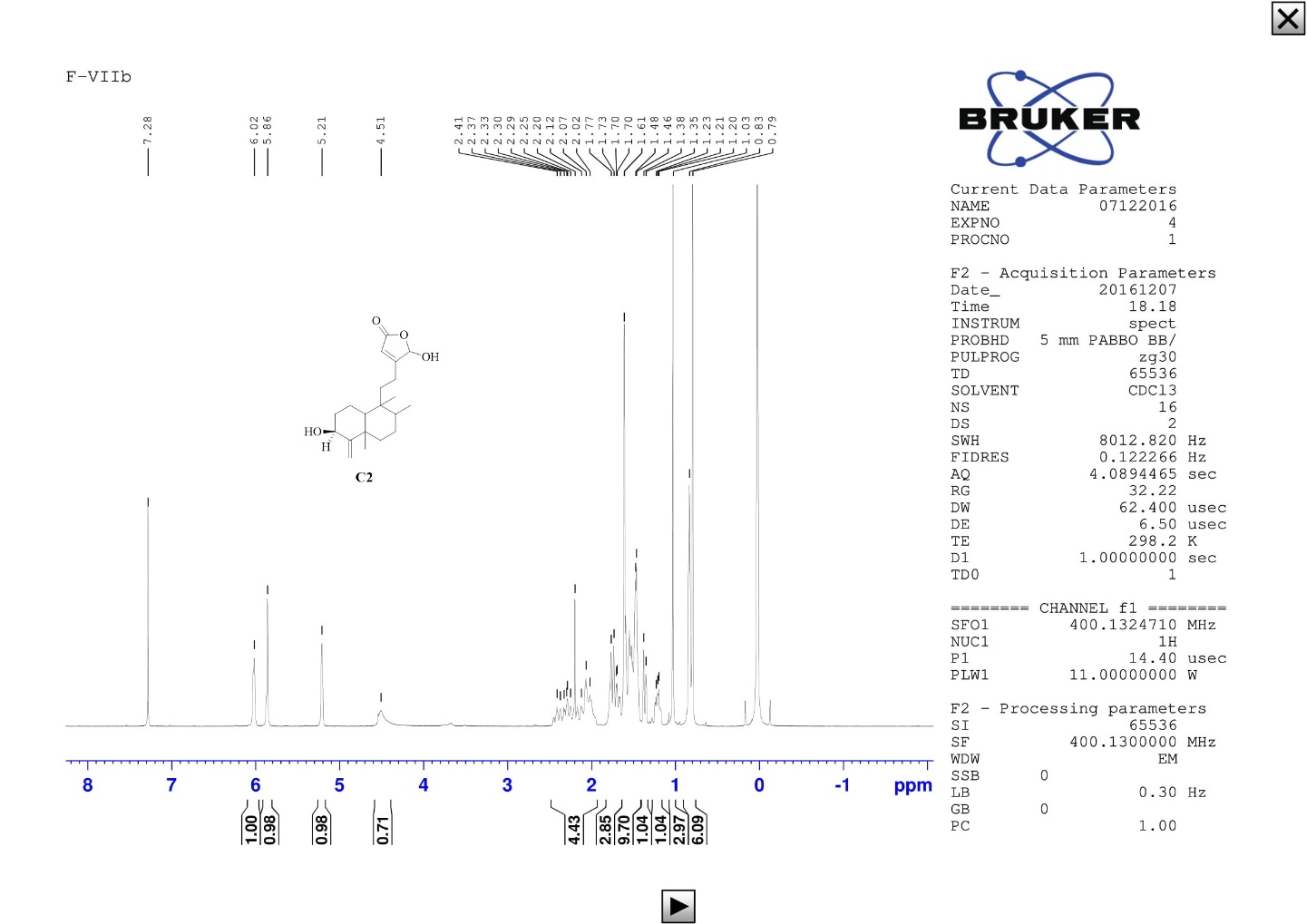
**

**
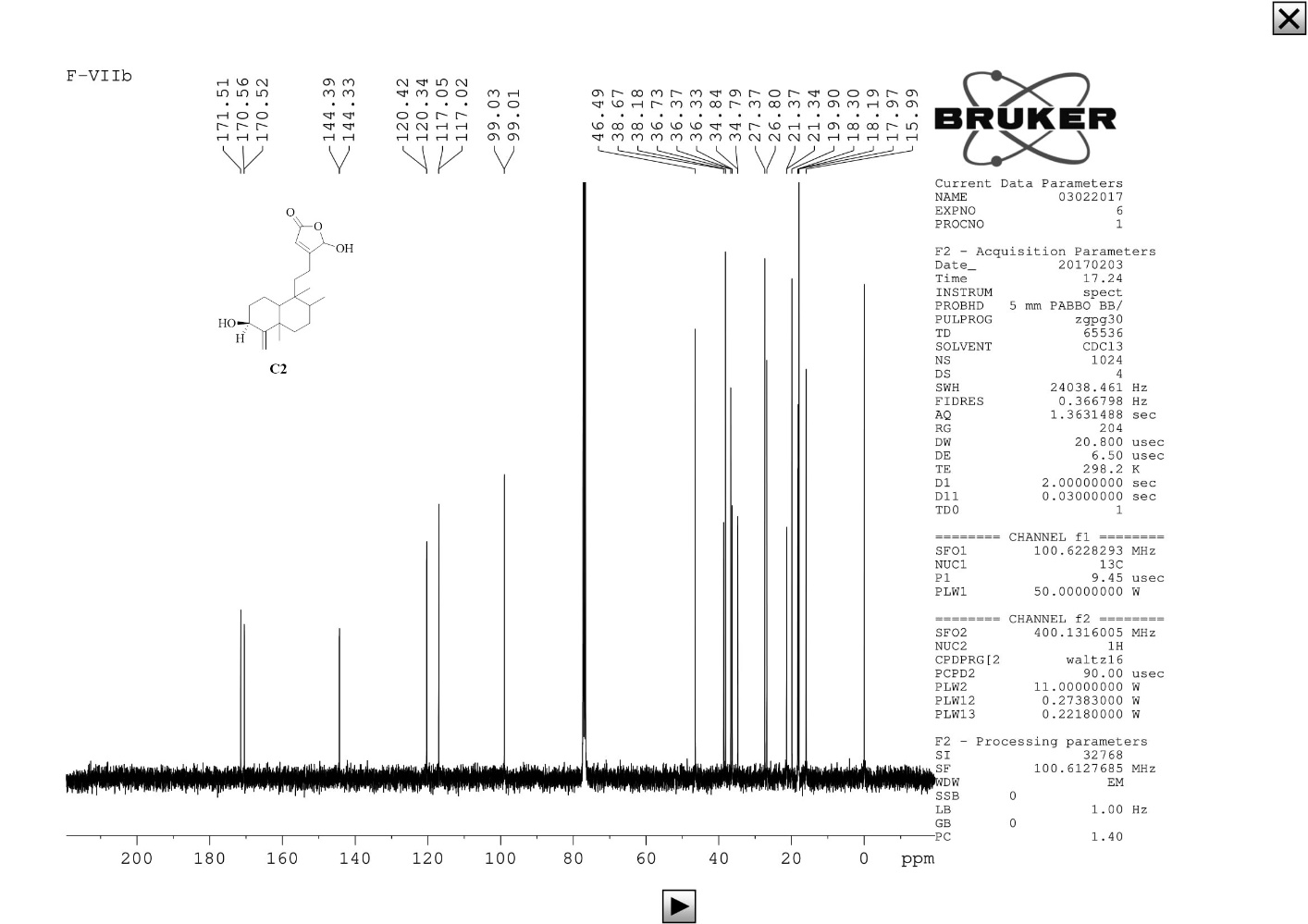
**


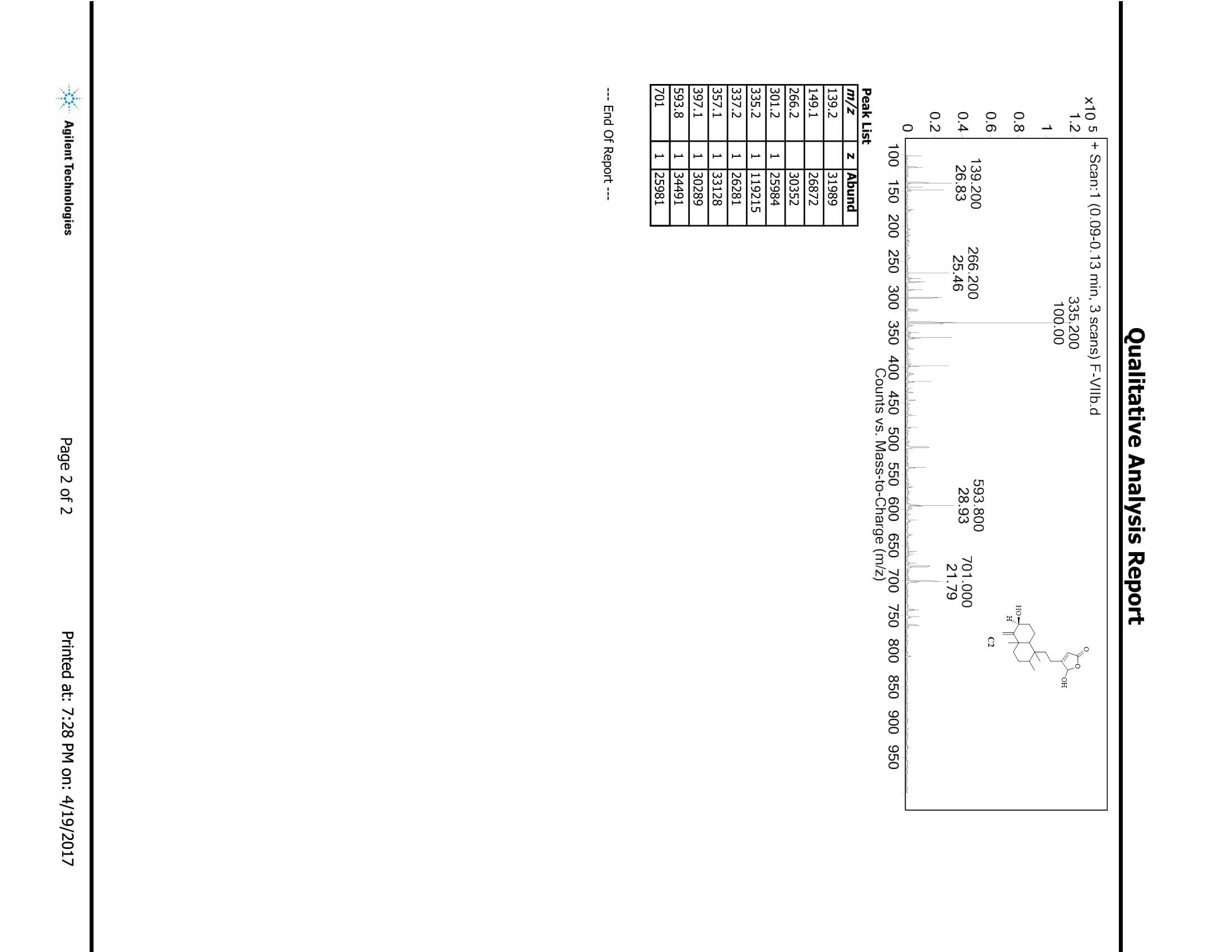


**Figure S1.** Spectral data of compound **6**. (**a**) ^1^H NMR (400 MHz, CDCl_3_); (**b**) ^13^C NMR (400 MHz, CDCl_3_); and (**c**) ESI-MS positive mode of compound **6**

**Chemical characterization of compound 6.**

**6** (*3β,16α-dihydroxy-cleroda-4(18), 13(14)Z-dien-15,16- olide*)^1^: White solid; Yield: 3.26 g; mp: 66-67 ˚C; UV (Methanol): λ_max_ 206; [α]_D_ = -3.30º (*c* = 0.25, CHCl_3_); ^1^H NMR (400 MHz, CDCl_3_): δ 6.02 (s, 1H), 5.86 (s, 1H), 5.21 (s, 1H), 4.51 (s, 1H), 2.25-2.41 (m, 3H), 2.20 (s, 2-OH), 2.02-2.12 (m, 2H), 1.70-1.77 (m, 6H), 1.61 (m, 2H), 1.35-1.48 (m, 1H), 1.20-1.23 (m, 1H), 1.03 (s, 3H), 0.83 (s, 3H), 0.79 (s, 3H) (Figure S1); ^13^C NMR (400 MHz, CDCl_3_): δ 171.51, 170.56, 144.39, 120.42, 117.05, 99.03, 99.01, 46.49, 38.67, 38.18, 36.73, 36.37, 34.84, 27.37, 26.80, 21.37, 19.90, 18.30, 18.19, 15.99 (Figure S2); ESI-MS *m/z*: [M]^+^ calcd. for C_20_H_30_O_4_: 334.21; found, 335.200 ([M]^+^ + [H]^+^, 100%, positive mode) (Figure S3); analysis (calcd., found for C_20_H_30_O_4_): C (71.82, 71.80), H (9.04, 9.04).

**
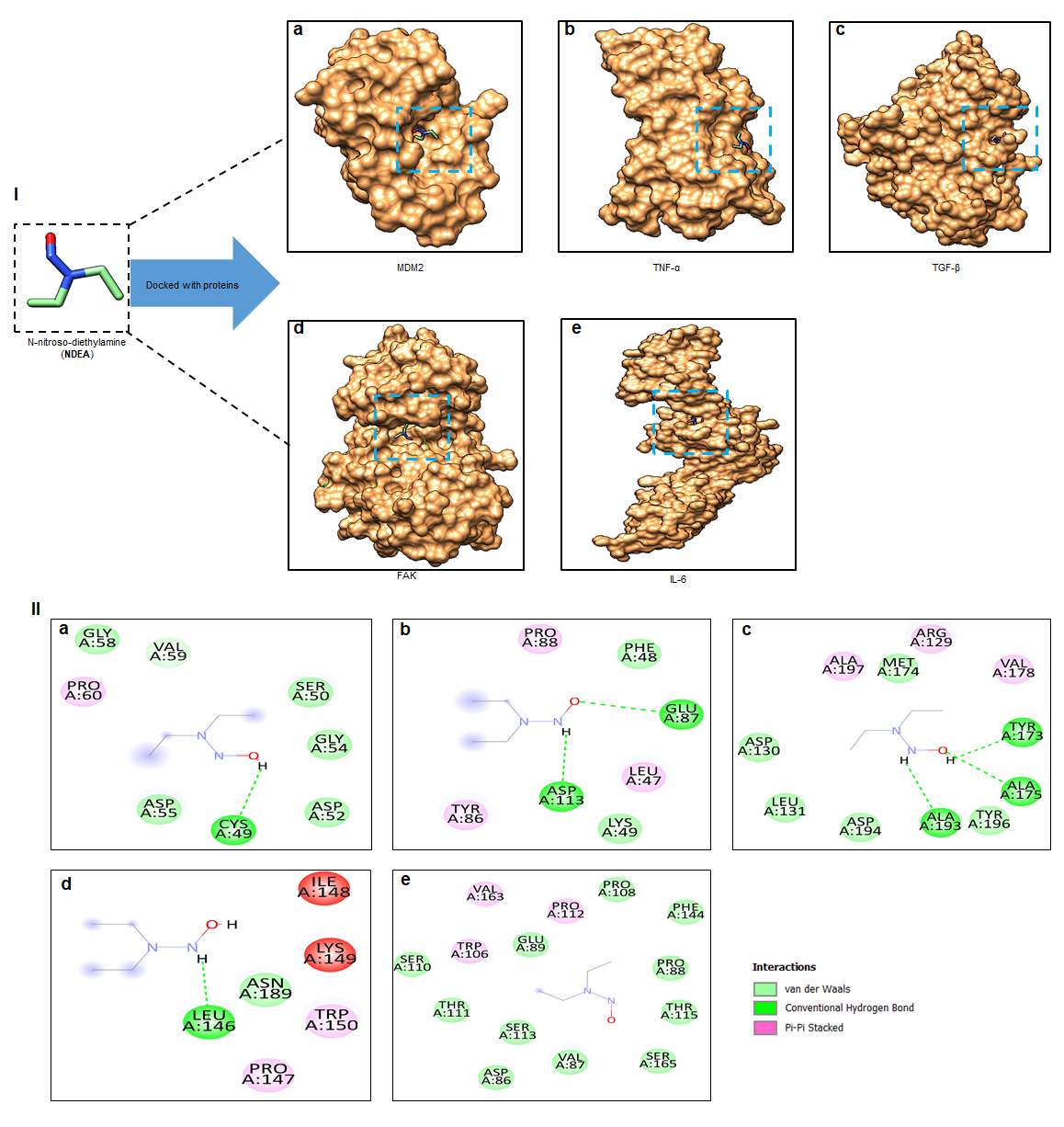
**

**Figure S2.** (I) Docking between NDEA and five proteins MDM2, TNF-alpha, TGF-beta, FAK, AND IL-6. (II) Hydrogen bond and hydrophobic interactions between NDEA and (**a**) MDM2, (**b**) TNF-alpha, (**c**) TGF-beta, (**d**) FAK, and (**e**) IL-6.

**
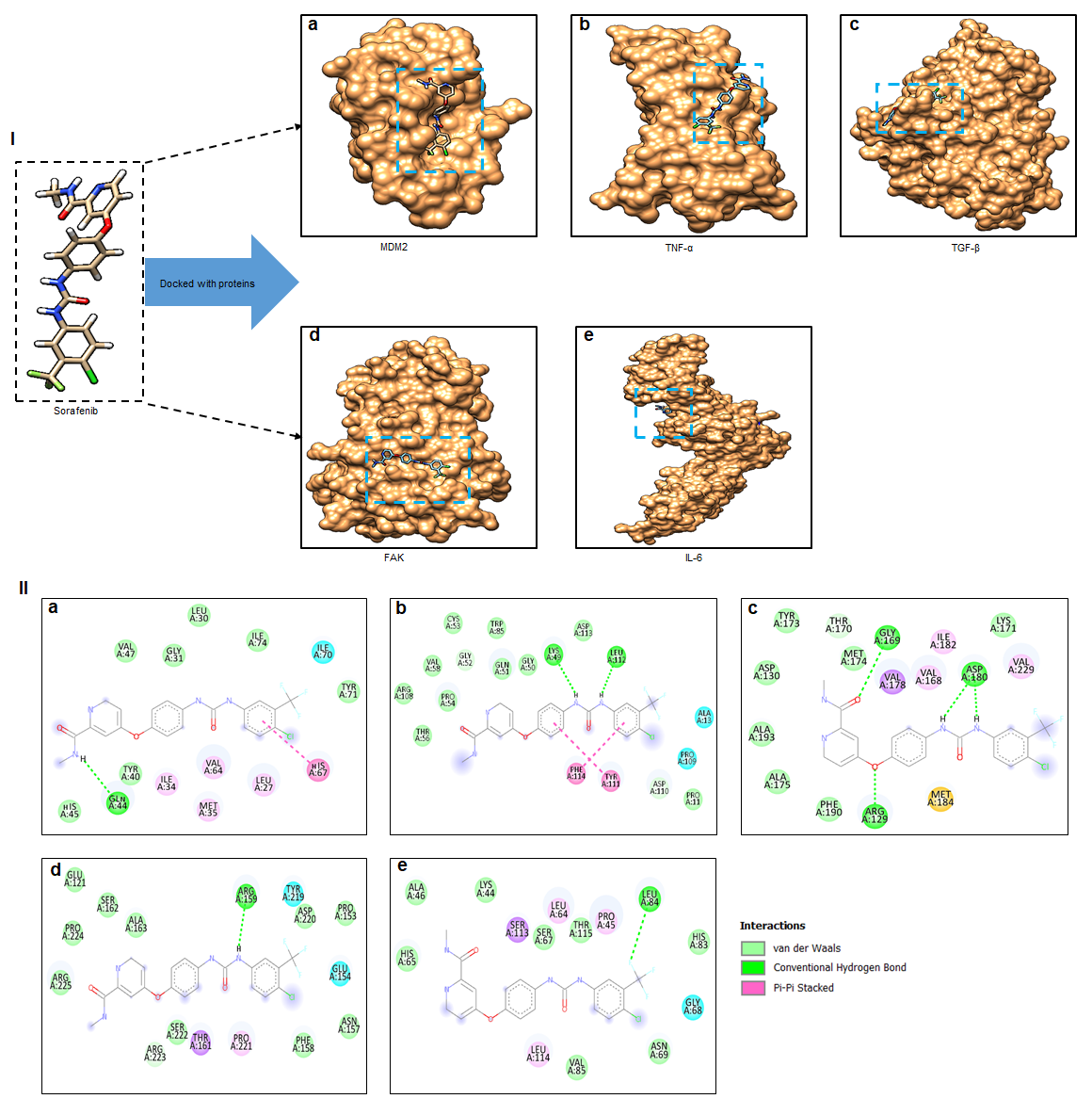
**

**Figure S3.** (I) Docking between Sorafenib and five proteins MDM2, TNF-alpha, TGF-beta, FAK, AND IL-6. (II) Hydrogen bond and hydrophobic interactions between Sorafenib and (**a**) MDM2, (**b**) TNF-alpha, (**c**) TGF-beta, (**d**) FAK, and (**e**) IL-6.

**Supplementary tables**

| **Protein Name** | **PDB ID** | **Active Sites** | **Ref.** |
| --- | --- | --- | --- |
| MDM2 | 1YCR | Gln59, Leu54, Phe55, Lys51, Gly58 | 2 |
| TNF-α | 2AZ5 | Leu57, Tyr59, Ser60, Gln61, Tyr119, Leu120, Gly121, Gly122, Tyr151 | 3 |
| TGF-β | 1RW8 | Ile211, Gly212, Lys232, Ser280, Tyr282, His283, Asp351 | 4 |
| FAK | 4Q9S | Ile428, Val436, Gln438, Ala452, Lys454, Leu501, Phe542, Arg545, Arg550 | 5 |
| AFP | - | Ser445, Arg452, Glu551 | 6 |
| IL-6 | 1N26 | Phe103, Lys105, Ser109, Val112, Gly114, Glu147, Tyr148, Ser149, Ser152, Lys154, Ser156, Gln158 | 7 |

**Table S1.** PDB ID’s and active sites of the proteins.

| **Sample** | **Percentage inhibition at different concentration (%)*** | | | | |
| --- | --- | --- | --- | --- | --- |
|  | **10 µg/mL** | **20 µg/mL** | **25 µg/mL** | **30 µg/mL** | **50 µg/mL** |
| Hep G2 cell line | | | | | |
| **1** | 15.78±1.37^c^ | 22.79±0.95^c^ | 33.03±0.42^c^ | 33.34±3.34^c^ | 36.11±4.65^c^ |
| **2** | 12.47±2.34^c^ | 19.23±3.95^c^ | 25.89±1.74^c^ | 30.21±2.94^c^ | 39.99±2.98^c^ |
| **3** | 30.18±3.98^b^ | 41.49±2.45^a^ | 43.11±4.78^b^ | 48.27±4.76^b^ | 56.27±4.57^b^ |
| **4** | 46.96±1.94 | 53.97±1.88 | 55.97±1.27 | 58.58±2.58 | 60.43±1.08^a^ |
| **5** | 20.48±2.90^c^ | 24.94±2.63^c^ | 43.65±1.96^b^ | 51.19±3.22^b^ | 53.89±3.06^b^ |
| **6** | 37.11±2.52^a^ | 47.81±3.65 | 50.04±2.94^a^ | 55.66±2.99^a^ | 60.97±3.77^a^ |
| **Sorafenib** | 49.96±1.51 | 55.60±1.37 | 63.40±2.62 | 71.90±1.46 | 78.67±0.95 |
| Huh 7 cell lines | | | | | |
| **1** | 11.74±0.83^c^ | 15.22±1.18^c^ | 17.54±2.02^c^ | 18.13±1.00^c^ | 22.45±1.48^c^ |
| **2** | 11.42±2.49^c^ | 13.14±1.43^c^ | 15.25±0.25^c^ | 17.71±0.80^c^ | 20.26±1.66^c^ |
| **3** | 10.01±2.14^c^ | 22.06±2.99^c^ | 34.83±1.46^c^ | 40.34±2.93^c^ | 44.03±1.48^c^ |
| **4** | 15.20±3.97^c^ | 22.98±2.63^c^ | 38.10±1.02^c^ | 43.72±0.80^c^ | 50.97±3.78^c^ |
| **5** | 21.66±4.96^b^ | 31.66±1.27^c^ | 33.40±1.43^c^ | 38.25±2.55^c^ | 45.45±3.56^c^ |
| **6** | 25.13±1.04^a^ | 29.21±0.15^c^ | 43.11±2.59^c^ | 46.68±3.99^c^ | 50.26±2.66^c^ |
| **Sorafenib** | 42.09±3.97 | 52.48±1.25 | 63.88±1.51 | 74.10±1.66 | 84.83±1.46 |

**Table S2.** Percentage inhibition and IC_50_ values of 1-6 against Hep G2 and Huh 7 cancer cell lines. *Values were presented as mean ± SD (*n* = 3) and analyzed by one-way analysis of variances followed by Tukey’s test, where ^a^*p* < 0.05; ^b^*p* < 0.01; ^c^*p* < 0.001 statistically significant when compared with the standard (sorafenib). ND: Not determined.

**References**

1. Ma, X. *et al.* Cytotoxic clerodane diterpenes from Polyalthia barnesii. *Phytochemistry* **37**, 1659–1662 (1994).
2. Adebayo, I. A., Arsad, H. & Samian, M. R. Methyl elaidate: A major compound of potential anticancer extract of Moringa oleifera seeds binds with bax and MDM2 (p53 inhibitor) In silico. *Pharmacogn. Mag.* **14**, S554–S557 (2018).
3. Zia, K. *et al.* Identification of potential TNF-α inhibitors: from in silico to in vitro studies. *Sci. Rep.* **10**, 20974 (2020).
4. Choodamani, B. *et al.* Synthesis, molecular docking and preliminary antileukemic activity of 4-methoxybenzyl derivatives bearing imidazo[2,1-b][1,3,4]thiadiazole. *Chem. Biodivers.* **18**, e20000800 (2021).
5. Daneial, B., Joseph, J. P. V. & Ramakrishna, G. Molecular dynamics simulation analysis of Focal Adhesive Kinase (FAK) docked with solanesol as an anti-cancer agent. *Bioinformation* **13**, 274 (2017).
6. Terentiev, A. A. *et al.* Modeling of three dimensional structure of human alpha-fetoprotein complexed with diethylstilbestrol: docking and molecular dynamics simulation study. *J. Bioinform. Comput. Biol*. **10**, 1241012 (2012).
7. Bharti, R. *et al.* Diacerein-mediated inhibition of IL-6/IL-6R signaling induces apoptotic effects on breast cancer. *Oncogene*  **35**, 3965–3975 (2015).
